# The role of CD74 in cardiovascular disease

**DOI:** 10.3389/fcvm.2022.1049143

**Published:** 2023-01-12

**Authors:** Qiu-Lin Li, Jing Tang, Ling Zhao, Amanguli Ruze, Xue-Feng Shan, Xiao-Ming Gao

**Affiliations:** ^1^State Key Laboratory of Pathogenesis, Prevention and Treatment of High Incidence Diseases in Central Asian, Department of Cardiology, The First Affiliated Hospital of Xinjiang Medical University, Ürümqi, China; ^2^Xinjiang Key Laboratory of Medical Animal Model Research, Ürümqi, China; ^3^Department of Clinical Laboratory, The First Affiliated Hospital of Xinjiang Medical University, Ürümqi, China; ^4^Clinical Medical Research Institute of Xinjiang Medical University, Ürümqi, China

**Keywords:** CD74, MIF, D-DT/MIF2, cardiovascular diseases, invariant chain

## Abstract

Leukocyte differentiation antigen 74 (CD74), also known as invariant chain, is a molecular chaperone of major histocompatibility complex class II (MHC II) molecules involved in antigen presentation. CD74 has recently been shown to be a receptor for the macrophage migration inhibitory factor family proteins (MIF/MIF2). Many studies have revealed that CD74 plays an important role in cardiovascular disease. In this review, we summarize the structure and main functions of CD74 and then focus on the recent research progress on the role of CD74 in cardiovascular diseases. In addition, we also discuss potential treatment strategies that target CD74. Our systematic review of the role of CD74 in cardiovascular disease will fill some knowledge gaps in the field.

## 1. Introduction

Leukocyte differentiation antigen 74 (CD74), also known as invariant chain (Ii), is a transmembrane glycoprotein that is not polymorphic. CD74 was initially identified only as a molecular chaperone of major histocompatibility complex class II (MHC II) molecules involved in exogenous antigen presentation. Subsequent studies found that CD74 also participates in the endogenous presentation of antigen through MHC I molecules (1, 2). Additionally, CD74 is also a high affinity membrane receptor of macrophage migration inhibitory factor (MIF) and d-dopachrome tautomerase (D-DT/MIF2). CD74 is also a functional receptor for tissue inhibitor of metalloproteinases-1 (TIMP-1) (3) and regulates the transport of angiotensin II type I receptor (AT1) (4). It activates a number of pathophysiological pathways when a complex is formed by CD44 and MIF/MIF2. For instance, phosphatidylinositol 3-kinase/protein kinase B (PI3K/Akt), adenosine monophosphate-activated protein kinase (AMPK), nuclear factor-κB (NF-κB), and extracellular signal-regulated kinase (ERK) pathways, that are involved in B cells differentiation and maturation, cell proliferation, and energy metabolism, etc. CD74 through these signaling pathways plays regulatory roles in the development and progress of various diseases, such as ischemic heart disease (5), atherosclerosis (6), hepatic fibrosis (7), immunological disorders, and cancers (1).

## 2. Structure and function of CD74

### 2.1. Structure of CD74

**FIGURE 1:**
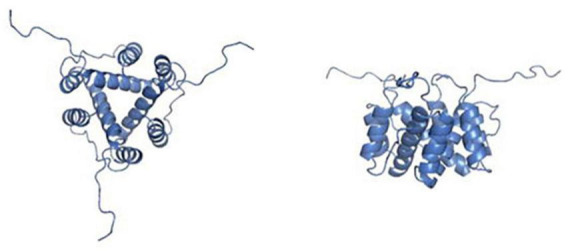
Pictures depicting the secondary, tertiary, and quaternary structures of leukocyte differentiation antigen 74 (CD74). The picture on the **left** shows the structural view from the top. The **right** picture shows a side view of the same homotrimer. Pictures adopted from Meza-Romero et al. with permission (No. 5392361035630) (8).

**FIGURE 2:**
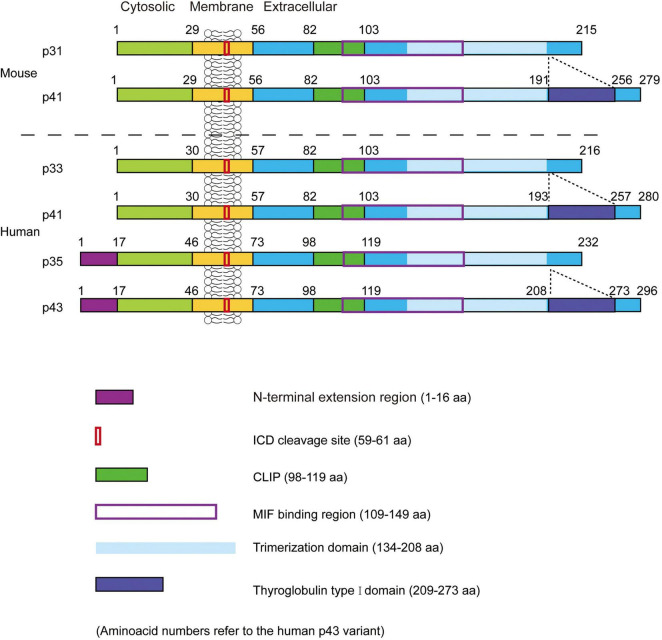
The structure of mouse and human leukocyte differentiation antigen 74 (CD74). Two isoforms (p31 and p41) in mice and four isoforms (p33, p35, p41, and p43) in humans. CLIP, the class II-associated invariant chain peptides; ICD, intracellular domains. Figure adopted from Schroder et al. with permission (No. 5390740929053) (19).

The molecular structure of CD74 is comprised of the *N*-terminal cytoplasmic region, the transmembrane region, and the C-terminal extracellular region. Structural models of CD74 are shown in Figure 1 (8). In human, CD74 is encoded by chromosome 5 with a gene length of roughly 9 kb and consisted of nine exons (9). There are four isoforms of human CD74, which can be divided into p33, p35, p41, and p43 according to their molecular weight (Figure 2), of which p33 (33 kDa) is the most common isoform and has a 216 amino acid sequence (10). There are two isoforms of CD74 in the mouse, p31 and p41, corresponding to human p33 and p41, respectively (11). In human, the start codons of p33 and p41 can be switched, allowing for an *N*-terminal extension of 16 amino acids to form other two isoforms, p35 and p43. Phosphorylation of this *N*-terminal extension domain is thought to be important for CD74-MHC II complex exiting from the endoplasmic reticulum and transporting to the endosomal compartment (12, 13). Additionally, in the gene sequence encoding the two longer isoforms (p41 and p43), an additional exon 6b (located between exon 6 and exon 7) can encode a cysteine rich domain containing 64 amino acids (209–273 amino acid residues refer to human p43 variant). This domain is called thyroglobulin type I domain, and it has high homology with thyroglobulin (9). This domain binds to cathepsin L and prevents it from degradation, allowing cathepsin L to remain active in the extracellular environment and regulate extracellular matrix degradation during inflammation (14). In addition, the class II-associated invariant chain peptides (CLIP), which contain the 98–119 amino acid residues (human p43 variant) of CD74 molecule, is a site that binds to MHC II and plays an important role in antigen presentation (15, 16).

### 2.2. Functions of CD74

#### 2.2.1. Function of antigen presentation

**FIGURE 3:**
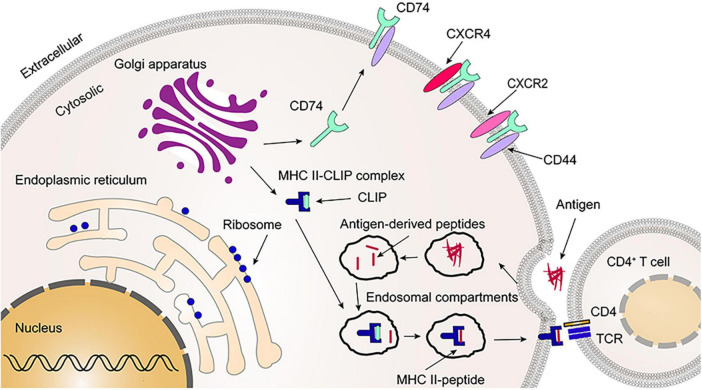
Primary functions of leukocyte differentiation antigen 74 (CD74). The CD74 is released to the cell surface following synthesis, folding, and trimerization (2–5%). Together with CD44, CXCR2 and CXCR4, CD74 serves as a receptor for migration inhibitory factor (MIF) and MIF2. As most of CD74 occupies the binding groove of major histocompatibility complex class II (MHC II), it prevents the binding of endogenous polypeptide molecules to MHC II in the endoplasmic reticulum. Afterwards, CD74 is partially cleaved, leaving the CLIP fragment that protects the binding groove of MHC II. Once the CLIP fragment is released, the exogenous peptide can be loaded into the binding groove of the MHC II and form the MHC II-peptide. Finally, CD4^+^ T cells recognize the MHC II-peptide on the cell membrane. CLIP, the class II-associated invariant chain peptides; TCR, T-cell receptor.

After synthesis in the endoplasmic reticulum, CD74 is first assembled into a homotrimer and then bound with three MHC II heterodimers (αβ) to form a nonamer (αβIi)_3_. CD74 occupies the binding groove of MHC II, which prevents the binding of endogenous peptide molecules to MHC II in the endoplasmic reticulum. The formation of CD74 homotrimer is related to transmembrane region and amino acid residues 134–208 of trimerization domain (human p43 variant) in *C*-terminal extracellular region, which is a protease resistant domain (10, 17–19). A recent study found that both the transmembrane region and the *C*-terminal trimerization domain can independently induce CD74 homotrimer formation, which is in the absence of either the transmembrane region or the *C*-terminal trimerization domain, but with less efficiency (18). Further, The study also found that no domain except the transmembrane region and the *C*-terminal trimerization domain could polymerize CD74 to form a trimer (18). Once the nonamers (αβIi)_3_ formed, it immediately leaves the endoplasmic reticulum and enters the Golgi apparatus. Subsequently, CD74 is hydrolyzed by proteases, leaving only the CLIP fragment to protect the binding groove of MHC II (19, 20). Inhibition of these proteases would prevent the antigen presentation and immune response in which MHC II is involved, while cell surface expression of MHC II is also inhibited (21, 22). Finally, the Class II accessory molecule DM (HLA-DM in human; H2-M in mouse) catalyzes the substitution of CLIP fragment by the antigenic peptide results in the formation of a mature MHC II-peptide complex that is released to the cell surface for recognition by CD4^+^T cells and initiates an immune response (Figure 3) (19, 23, 24). CD74 has also been shown to be involved in MHC I presenting antigens, which in turn activates CD8^+^T cells, causing MHC I-mediated cytotoxic T-cells responses (2). In addition, it was found that the number of CD4^+^ T cells in thymus and peripheral blood was greatly reduced in CD74^–/–^ mice but had a more CD8^+^ T cells compared to wild-type (WT) control mice (6).

#### 2.2.2. Receptor function

MIF is a multifunctional inflammatory cytokine, composing of 115 amino acid residues. It is a highly conserved protein with a molecular weight of about 12.5 kDa. MIF has many functions, such as pro-inflammation, chemotaxis and regulating cell proliferation, angiogenesis, and fibrosis (25). MIF also possesses oxidoreductase and tautomerase activities, but the role of MIF enzyme activity is still not very clear, and some studies have found that it may be related to immune function (26). MIF2 shares similar amino acid sequence, structure and biological activity with MIF and MIF2 also has tautomerase activity similar to MIF. MIF2 consists of 117 amino acid residues with a molecular weight of 13 kDa (27). The genes of MIF and MIF2 are within 80 kb from each other in mouse and human genomes. The amino acid sequence homology of human MIF and MIF2 is 34%, and that of mouse MIF and MIF2 is 27% (27). Like MIF, MIF2 also forms a homotrimer structure, and its overall topology and trimeric structure are similar to MIF (28).

Leng et al. first found that CD74 is a high affinity membrane receptor for MIF. The extracellular domain between 109 and 149 amino acid residues (human p43 variant) of CD74 binds to MIF (Figure 2), and one MIF homotrimer binds three CD74 homotrimers to form a dodecamer (29). Merk et al. found that MIF2 is also a high affinity ligand for CD74 through competitive binding experiments, and MIF2 diminishes the binding of MIF to the extracellular domain of CD74 in a dose-dependent manner. The binding rate of MIF2 to CD74 is approximately three times higher than MIF, but the dissociation rate is 11 times faster than MIF (27). Different from MIF and CD74 binding to form a dodecamer, one CD74 homotrimer binds to only one MIF2 homotrimer to form a hexamer (30).

CD44 is a transmembrane glycoprotein with well-known kinase-activating properties, it participates in MIF and MIF2 signaling together with CD74. CD44 consists of three components: the *N*-terminal extracellular region (amino acid residues 1–270), the transmembrane region (amino acid residues 271–291), and the C-terminal intracellular region (amino acid residues 292–361) (31). A recent study documented the formation of CD74/CD44 complexes by immunoprecipitation, and further observed that neither CD74^+^CD44^–^ nor CD74^–^CD44^+^ cells could activate the MIF signaling pathway. These findings suggest that CD74 is required to work together with CD44 to mediate the MIF/MIF2 signaling pathway (30). Due to alternative splicing, CD44 has subtypes with different molecular weight and size, but only the full length of CD44 can assist CD74 in MIF signaling (32). Similarly, CD74/CD44 is also involved in MIF2 signaling (27). Besides, CD74 can form complexes with CXCR2, CXCR4 or CXCR7 to participate in MIF signaling (Figure 3) (30).

MIF binds to the CD74/CD44 complex to activate Src-family protein kinases, and then activate extracellular signal-regulated kinase-1/2 (ERK1/2), mitogen-activated protein kinase (MAPK), PI3K-Akt cascade signaling, and inhibit p53. These signaling pathways are involved in regulation of inflammation, cell proliferation, apoptosis and survival, prostaglandin E2 production and tumorigenesis (29, 30, 33–35). MIF-CD74/CD44 can also activate the NF-κB pathway. It has been found that CD74 lacking the extracellular region can still activate the NF-κB pathway and induce differentiation and maturation of B lymphocytes (36, 37), suggesting that NF-κB activation may be mediated by the CD74 intracellular domains (CD74-ICD) (38). CD74-ICD is formed through the regulated intramembrane proteolysis (RIP), which then enters the nucleus to activate NF-κB (39). In addition, soluble CD74 (sCD74) is a membrane truncated protein that has been shown to be associated with a variety of diseases, such as autoimmune liver disease, acute respiratory distress syndrome, burn injury, myocardial ischemia-reperfusion (I/R) injury, and myocardial fibrosis (40–44). sCD74 has been shown to bind MIF and regulate its activity (29, 45). There is a 6 kDa difference in molecular weight between full-length CD74 and truncated sCD74. Interestingly, the molecular weight of CD74-ICD is also known to be 6 kDa. Therefore, it is speculated that the two truncated short segments of CD74, sCD74, and CD74-ICD, may be generated simultaneously by the RIP process (40).

### 2.3. Expression of CD74

It appears that those cells expressing MHC II also express CD74, including B-cells, monocytes, Langerhans cells, dendritic cells, and thymic epithelial cells (46). Whereas, some cells such as macrophages, epithelial and endothelial cells, podocytes, vascular smooth muscle cell, cardiac fibroblasts, and cardiomyocytes, even without MHC II, also express CD74, which imply the existence of a mechanism for translocation of CD74 to the cell surface during development independent of MHC II (1, 47–52). However, some cell types do not express CD74, such as neutrophils or the cell lines HEK293 and HeLa (53). CD74 predominantly exists in cells, and only 2–5% of CD74 is expressed on the cell surface independent of MHC II (46). Due to rapid endocytosis, the residence time of CD74 on the cell surface is very short, and the half-life is less than 10 min, with 3 × 10^3^ CD74 molecules were endocytosed every minute. However, owing to its rapidly renewal, about 4 × 10^6^ CD74 molecule can be expressed cumulatively on the cell surface daily (54).

In the context of CD74 expressed in a verity of cardiac cell types and immune cells, manifestation, pathophysiological impact and potential clinical significance of CD74 and MIF family proteins and potential mechanisms or signaling pathways involved will be discussed under different pathological settings.

## 3. The role of CD74 in cardiovascular diseases

### 3.1. The role of CD74 in atherosclerosis

The pathologic basis of coronary atherosclerotic heart disease is the chronically long-term growth of atherosclerotic plaques in the coronary vessel wall, which leads to the lumen stenosis, blood flow limitation and plaque rupture with acute thrombotic occlusion. Chronic inflammation of the vessel wall, characterized by the infiltration of circulating immune cells, is a key factor in the development of atherosclerotic lesions (55). Cytokines are released at the site of tissue injury or infection and are regulators of adaptive immune response and inflammatory response. MIF is a pro-inflammatory regulator of many acute and chronic inflammatory diseases and can modulate the development of atherosclerotic lesions (56). The role of MIF in atherosclerosis has been intensively investigated. MIF is expressed in atherosclerotic plaques, which is highly expressed in unstable plates (56–58). Blocking MIF can help vulnerable plaques transition to a more stable and less prone to rupture state (55). MIF has a chemokine-like function that activates CXCR2 and CXCR4, and thus can recruit monocytes and T lymphocytes to promote atherosclerosis (57, 59, 60).

**FIGURE 4:**
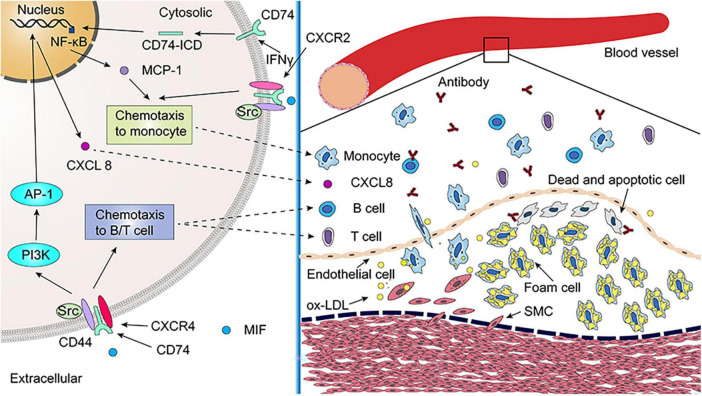
The role of leukocyte differentiation antigen 74 (CD74) in atherosclerosis. Migration inhibitory factor (MIF) promotes monocyte chemotaxis *via* CXCR2/CD74/CD44 complex. The secretion of monocyte chemoattractant protein-1 (MCP-1) is promoted by inhibits atherosclerosis by reducing interferon-gamma (IFNγ) inducing CD74/NF-κB. The MIF/CXCR4/CD74/CD44 signaling pathway promotes B-cell and T-cell chemotaxis, which releases antibodies that neutralize ox-LDL and promote the clearance of dead and apoptotic cells in atherosclerotic lesions. Additionally, MIF promotes CXCL8 expression through CXCR4/CD74/CD44, which plays an important role in atherosclerosis. As of yet, it is unclear whether MIF2 contributes to atherosclerotic lesions. SMC, smooth muscle cell; ox-LDL, oxidized low density lipoprotein.

Bernhagen et al. observed that atherogenic or inflammatory monocyte recruitment induced by MIF is not only CXCR2 dependent but also involves the its receptor, CD74. The formation of CXCR2/CD74 complexes was discovered by co-immunoprecipitation, indicating that MIF transmits signals through functional CXCR2/CD74 complexes (Figure 4) (60). CD74 lacks a domain that transduces intracellular signals. However, CD74 can signal by recruiting CD44 and Src family protein kinases, thereby generating chemotaxis and recruiting monocytes. MIF-induced leukocyte chemotaxis *via* CXCR2/CD74 was further confirmed by blocking both CXCR2 and CD74 that significantly impaired the ability of MIF to induce monocyte infiltration (60).

In T-cells, MIF can also quickly trigger the activation of the classical JNK/c-Jun/AP-1 pathway through the CXCR4/CD74/Src/PI3K axis, which induces the activation of cytokine CXCL8 or IL-8 mediated signaling pathway and promoting inflammatory processes. CXCL8 is an inflammatory factor that has been linked to the progression of atherosclerosis (61). Whether MIF2 is involved in the regulation of the atherosclerotic process remains unclear as MIF2 does not have an *pseudo-(E)LR* motif like MIF and cannot activate CXCR2 (45, 58, 62). Deficiency of T-cells that mediate adaptive immune responses can reduce atherosclerotic plaques by 80%, and CD4^+^T cells are the major subtype of T-cells engaged in atherosclerosis. Their activation requires antigen presentation by antigen presenting cell (APC), and MHC II mediate this process (63). CD74, as a molecular chaperone of MHC II molecules, is involved in the folding of MHC II, intracellular transport and loading of antigen peptides (10). Sun et al. observed that atherosclerosis was aggravated in LDLR^–/–^ mice after high-fat diet feeding while the degree of atherosclerosis was lower in hybrid mice with both LDLR and CD74 deficiency, which was associated with an impaired adaptive immune response to disease-specific antigens (6). In term of mechanism, CD74 deficiency impairs APC antigen presentation and T-cell activation, which reduces atherosclerosis (6).

In addition, MIF also promotes B lymphocyte survival by activating the CD74-CD44 receptor complex (64). Primary B-cells from WT mice incubated with anti-CD74 antibody or AMD3100 to block CD74 or CXCR4, respectively, both inhibited the chemotactic response of B-cells to MIF, suggesting that MIF exerts a chemotactic effect on B-cells *via* both CD74 and CXCR4 (65). Circulating B-cell numbers are reduced in MIF-deficient atherosclerotic mice accompanied by defective B-cell development (66). Notably, MIF/CD74 signaling pathway induces B-cell maturation and survival independently of its chaperone activity (38, 67, 68). Oxidized low density lipoprotein (ox-LDL) can be phagocytized rapidly by macrophages and smooth muscle cells and exhibits the property of monocyte chemotaxis. It is a major factor in the formation of foam cells. B-cells play a protective role against atherosclerosis by producing natural IgM antibodies, which are used to neutralize ox-LDL and to promote the clearance of apoptotic and dead cells in atherosclerotic lesions (6, 69).

There is evidence for a role of CD74 in atherosclerosis. CD74 expression was elevated in carotid plaques and peripheral blood mononuclear cells (PBMCs) of patients with carotid stenosis, and correlated with carotid intima-media thickness in subjects without clinical cardiovascular disease (49). A very recent study also found that in human carotid plaques, CD74 expression is lesion-dependently increased and is associated with necrotic core formation and plaque rupture, clinical symptoms, macrophage apoptosis, ferritin, and thrombin receptors (70). Moreover, CD74 levels were inversely correlated to high-density lipoproteins and statin treatment, and positively correlated to triglycerides (70). This study suggests that CD74 in apoptotic macrophages is linked to inflammation and thrombosis in progression of human atherosclerotic plaques, lipid metabolism, and clinical manifestation in atherosclerosis. Surface CD74 in apoptotic macrophages and ferritin production induced by oxidized lipids may contribute to inflammation and plaque vulnerability in atherosclerosis. Thus, CD74 may be a potential therapeutic target for atherosclerosis by reducing inflammatory response, which require further preclinical study to validate.

### 3.2. The role of CD74 in ischemic heart disease

Many experimental studies have revealed protective effect of MIF family proteins and its receptor in the animal model of ischemic heart disease. Qi et al. observed a larger infarct size in CD74^–/–^ compared with MIF^–/–^ mice after 15 min ischemia followed by 30 min reperfusion, which was approximately about twice that of MIF^–/–^ mice (71). This observation may imply a converged/central role of CD74 in mediating cardio-protection. In other word, there may be more factors rather than only MIF through CD74 involving in the downstream signaling transduction. Notably, the expression levels of MIF, CD74 and CD44 in the infarct zone were significantly upregulated in both the acute (1 day) and subacute (5 days) phases of the MI mouse model, further supporting a key role of MIF *via* CD74 in this disease condition (5).

**FIGURE 5:**
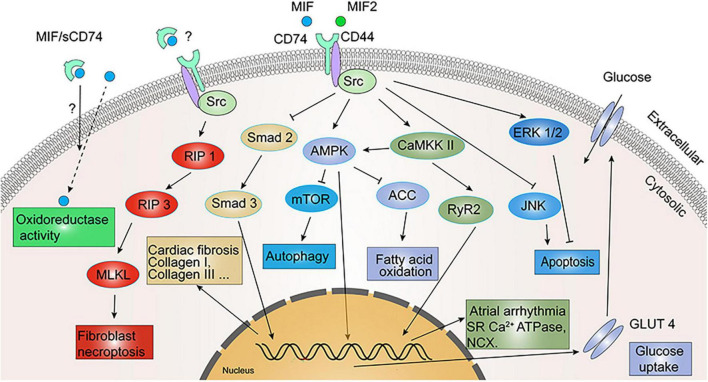
Signaling cascades in cardiovascular diseases involving migration inhibitory factor/leukocyte differentiation antigen 74 (MIF/CD74) and MIF2/CD74. MIF exerts myocardial protection by inhibiting c-Jun N-terminal kinase (JNK) and activating extracellular signal-regulated kinase (ERK) 1/2-mediated cardiomyocyte apoptosis. MIF/CD74 and MIF2/CD74 reinforce glucose uptake and utilization by activating adenosine monophosphate-activated protein kinase (AMPK) to promote GLUT4 expression, and inhibit fatty acid oxidation by inhibiting ACC. MIF/CD74 promotes sarcoplasmic reticulum (SR) Ca^2+^ ATPase and Na^+^/Ca^2+^ exchanger (NCX) expression and activation *via* CaMKK II/RyR2 to regulate atrial arrhythmia. MIF/CD74 inhibits myocardial fibrosis through inhibition of Smad 2/Smad 3 and promotes fibroblast necroptosis through regulated intramembrane proteolysis 1 (RIP 1)/RIP 3/MLKL. Oxidoreductase activity of MIF also attenuates myocardial hypertrophy and I/R injury oxidative stress, while sCD74 appears to enhance this activity.

Both MIF and MIF2 are highly expressed in murine cardiomyocytes and secreted by the heart under ischemic stress (52, 71, 72). MIF released from cardiomyocytes activated the CD74/CD44/AMPK signaling pathway, which leads to an increased glucose uptake by cardiomyocytes and thus protects the heart (52, 73–75). Likewise, MIF2 was also able to protect ischemic heart from dysfunction and necrosis by activation of the metabolic stress enzyme AMPK, which was mediated by a CD74/CaMKK II-dependent mechanism (71). In addition, MIF also exerts cardio-protection by activating ERK1/2 and inhibiting c-Jun N-terminal kinase (JNK) induced apoptosis in cardiomyocytes (76), anti-oxidative stress (44, 77, 78) or elevating oxidoreductase activity (Figure 5). It is worth to note that all these protective effects were achieved under a short ischemic duration.

However, open-heart surgery with cardiopulmonary bypass (CPB) causes ischemia-reperfusion of the heart, which triggers inflammatory reactions and leads to organ dysfunction in patients. There is an early study reported that MIF levels after CPB correlated with multiple organ dysfunction including hematological, circulatory and pulmonary global organ dysfunction (79). Thereafter, several clinical studies further investigated dynamic changes and clinical significance of MIF family proteins during cardiac surgery with CPB and reported opposite results. They found that serum levels of MIF and MIF2 significantly increased intraoperatively, while sCD74 decreased correspondingly (44). Interestingly, the time-change curves of circulating MIF and MIF2 levels are similar, but the serum levels of MIF are approximately five times higher than MIF2 (44). The peak value of MIF release is negatively related to post-operative atrial fibrillation and acute kidney injury, whereas the level of MIF2 is opposite of MIF and intraoperative MIF2 levels are related to the increased incidence of post-operative atrial fibrillation and pneumonia (44, 77). Moreover, decreased sCD74 levels during myocardial reperfusion were negatively correlated with MIF levels and circulating sCD74/MIF complexes can be detected in 50% of patients subjected to cardiac surgery with CPB, which acts as a protective agent against ischemic injury by enhancing the oxidoreductase activity of MIF (44).

The sCD74 was recently identified in autoimmune liver disease and played a role in regulating MIF signaling activity (40). There are other studies reported that sCD74/MIF complex was able to enhance the oxidoreductase activity of MIF, thereby protecting the heart and reducing the risk of acute kidney injury induced by oxidative stress after cardiac surgery (44, 77, 78). These findings emphasize the anti-oxidative action of MIF owing to its oxidoreductase activity. However, in comparison to MIF, MIF2 is deficient in oxidoreductase activity (44). Besides AMPK activation, MIF improves ischemic myocardial protection also *via* CXCR2/CD74 in cardiac resident cells, while it adversely affects CXCR2-expressing inflammatory cells (53). Recombinant MIF induced rapid LV systolic dysfunction, an adverse effect not associated with CD74 (80). Surprisingly, recombinant MIF2 did not adversely affect cardiac systolic function or heart rate (71). These findings suggest that MIF2 lacks the *pseudo-(E)LR* motif and fails to induce inflammatory cell infiltration and it is therefore more selectively protective in ischemic myocardium.

In summary, albeit MIF and MIF2 shares partial sequence and structural homology, interaction between MIF, MIF2, and CD74 may be independent. Especially, a verity of cardiac cells and immune cells express MIF family proteins and their receptor, under certain diseased settings, MIF, MIF2, CD74, and sCD74 may exert diverse pathophysiological effect owing to serval reasons. *First*, CD74 participates in T-cell antigen presentation independently of MIF and MIF2, and T-cells play an important role in atherosclerosis. It has been reported that the number of CD4^+^ T-cells in thymus and peripheral circulation is greatly reduced in CD74-deficient mice, further confirming the role CD74 plays in T-cell development. *Second*, CD74 is also a functional receptor for TIMP-1 and regulates the transport of AT1. *Third*, although CD74 is involved in some of the roles of MIF together with CXCR2/4, MIF can act through CXCR2/4 alone. *Fourth*, MIF is also able to bind with CXCR7 to activate PI3K-Akt pathway independent of CD74 (81). In addition, MIF also possesses oxidoreductase and tautomerase activities, of which their functions still need to be further explored. Finally, a truncated fragment of CD74, sCD74, is released extracellularly to neutralize MIF and regulate MIF oxidoreductase activity.

### 3.3. The role of CD74 in pathological myocardial hypertrophy

Hypertension increases cardiac afterload and the heart enhances contractility to overcome peripheral resistance and maintains effective cardiac output, which results in left ventricular hypertrophy. Under pressure-overload conditions, MIF^–/–^ mice showed more severe myocardial hypertrophy, systolic dysfunction, and myocardial fibrosis than WT mice (82, 83). This may be attributed to the fact that MIF can activate autophagy through AMPK-mTOR signaling (Figure 5), thereby alleviating the degree of cardiac hypertrophy in mice under stress loading conditions (82). Moreover, the inherent oxidoreductase activity of MIF also reduces the degree of myocardial hypertrophy under pressure-overload by limiting the oxidative stress of cardiomyocytes (84). MIF2 myocardial-specific knockout mice undergone transverse aortic constriction (TAC) developed cardiac systolic dysfunction faster than WT mice, along with more severe cardiac dilatation and pulmonary edema (84). It is worth noting that CD74 expression was significantly increased in both WT and MIF^–/–^ mice under pressure-overload conditions, suggesting that activation of CD74 may engage in the cardio-protection of MIF and MIF2 (83), but there is still a lack of relevant direct evidence.

### 3.4. The role of CD74 in other diseased hearts and pathological settings

Diabetic cardiomyopathy is featured by ventricular dilatation, cardiac remodeling (hypertrophy, interstitial fibrosis), loss of ventricular compliance, diastolic dysfunction, and finally systolic dysfunction. Chen et al. reported elevated plasma MIF levels in patients with diabetes mellitus (type 2 diabetes mellitus, T2D) (85). They then established a T2D model in WT and CD74^–/–^ mice by injection of streptozotocin (STZ) together with high-fat diet feeding and found that T2D mice developed obvious global metabolic disorder, cardiac remodeling, contractile dysfunction, myocyte apoptosis, pyroptosis and ferroptosis, and mitochondrial dysfunction. Knockdown of CD74 attenuated the above-mentioned adverse reactions induced by T2D without affecting the overall metabolic disorder (85). Moreover, acute ethanol injury and lipopolysaccharide (LPS) injury can lead to contractile dysfunction, cellular ultrastructural changes, inflammation, and apoptosis in the mouse heart. CD74 knockout can attenuate acute ethanol- and LPS-induced injury through AMPK-mTOR-mediated autophagy regulation (51, 86).

In a hyperoxic lung injury model, MIF- or CD74-deficiency and CD74 inhibition treatment results in worsen lung injury and poor survival, and inhibition of CD74 in primary murine lung endothelial cells (MLECs) abrogated the protective effect of MIF, suggesting endothelial CD74 mediates MIF protection in lung injury (87). In addition, interaction between rMIF and CD74 expressed on the surface of alveolar macrophages induces p44/p42 MAPK activation and inflammatory chemokine release, resulting in the accumulation of neutrophils in the alveolar space. This result suggests that alveolar macrophage-derived CD74 may mediate neutrophilic lung inflammation and acute lung injury (88).

Increased CD74 expression levels in circulating PBMCs mainly in CD4^+^ T cells, monocytes and dendric cells has been reported in human ischemic stroke patients (89). The number of CD74^+^ cells in blood correlated strongly with infarct size and neurological outcomes. However, the manifestation, exact action and clinical significance of CD74 expressed in a verity of different cell types under cardiac pathological settings is still required for further explorations.

### 3.5. Anti-fibrotic effect of CD74

Heinrichs et al. found in liver fibrosis disease that MIF exerted an anti-fibrotic effect by inhibiting PDGF-induced hepatic stellate cell migration and proliferation, which was promoted by CD74 through phosphorylation of AMPK (7). In mouse pressure-overload (TAC) model, the degree of fibrosis was four times higher in MIFKO mice than in WT mice (83). The combination of exogenous sCD74 and recombinant MIF could inhibit the MIF/CXCR4 axis, and induce a molecular switch from MIF-mediated transduction through pro-fibrotic CXCR4/Akt to anti-fibrotic CD74-mediated cell death, phosphorylation of protein kinases RIP 1 and RIP 3 (Figure 5), and finally necroptosis in the fibroblast (43). Endogenous CD74 also stimulates the antifibrotic effect of sCD74/MIF complex. Unlike the influence of sCD74/MIF in fibroblasts, combination of sCD74/MIF did not induce cardiomyocyte death (43). By measuring serum levels of sCD74 and MIF in patients with heart failure or MI and in healthy subjects, researchers found that the ratio of serum sCD74/MIF was only one-tenth that of normal controls in heart failure patients, and one-fifth that of normal controls in MI patients, and that the increased sCD74/MIF ratio would enhance the depletion of myofibroblasts in infarcted scars and alleviate reactive fibrosis (43). Ma et al. reported that rMIF2 treatment for mouse cardiac fibroblasts reduced TGF-β-induced Smad-2 phosphorylation and formation of extracellular matrix, thereby attenuating myocardial fibrosis. They then isolated cardiac fibroblasts from CD74^–/–^ mice and stimulated with rMIF2 and found that rMIF2 failed to inhibit TGF-β-induced Smad-2 phosphorylation, indicating that CD74 receptor is required for mediating antifibrotic effects of MIF2 in fibroblasts (84).

### 3.6. Role of CD74 in arrhythmia

A higher circulating level of MIF was reported in patients with atrial fibrillation (90). MIF may be a key factor in atrial arrhythmogenesis by regulating *T*-type and *L*-type calcium channels in atrial myocytes (91, 92). Additional evidence suggests that intraoperative serum MIF levels during myocardial reperfusion are independently associated with a lower risk of post-operative atrial fibrillation (44). MIF-treated atrial myocytes presented a higher calcium transient, increased sarcoplasmic reticulum calcium content, Ca^2+^ leakage, and Na^+^/Ca^2+^ exchanger (NCX) efflux rate. Whereas blocking CD74 abolished these effects of MIF (93). Likewise, MIF-treated atrial myocytes had augmented potassium currents, and blocking CD74 diminished such effect. In whole animals, MIF treatment in mice exhibited increased spontaneous atrial ectopic beats, but treatment with both MIF and anti-CD74 antibodies tended to have less atrial ectopic beats and atrial fibrosis (93). Moreover, treatment with anti-CD74 antibody only partially reversed MIF-induced atrial arrhythmias in mice (93). The potential underlying mechanism is related to expression of ATPase, NCX, ryanodine receptor 2 (RyR2) phosphorylation and CaMKII-RyR2 signaling activation triggered by MIF, which can be attenuated by blocking CD74 (Figure 5) (93). In additional, bioinformatic analysis of the myocardium of patients with arrhythmogenic right ventricular cardiomyopathy (ARVC) versus normal controls revealed an immune-related hub module, which Protein-Protein Interaction Network analysis identified CD74 as one of the most important hub genes (94). This information suggests that MIF/CD74 signaling is a potential therapeutic intervention for arrhythmia.

## 4. Treatment strategies that target CD74

### 4.1. Small molecule inhibitors of cathepsin S

Cathepsin S is a cysteine protease of the papain family which modulates the antigen presentation of MHC II and is active in secreted form (95). It is reported that cathepsin S is mainly expressed in APCs such as B-cells, macrophages, and dendritic cells (96). MHC II molecules are loaded with antigenic peptides after degradation of CD74 and release of CLIP. In APCs, cathepsin S gene knockout or inhibition by small molecule compounds reduces the degradation of CD74 and prevents the binding of MHC II to antigen peptides, thus reducing antigen presentation to CD4^+^T cells (96). Studies have found that the increase of serum cathepsin S level was related to the aggravation of coronary artery stenosis, carotid artery thickening, blood pressure increase, and abnormal vascular endothelial function (97). However, it is not related to the increased risk of cardiovascular events in patients with stable coronary heart disease (98). Plasma cathepsin S is significantly elevated in patients with unstable angina, and the higher level of cathepsin S suggests the existence of vulnerable plaques (99). Endogenous cathepsin S plays an important role in atherosclerotic plaque instability and plaque rupture (100). Cathepsin S may participate in the progression of atherosclerosis through macrophage mediated phagosomes (101). Treatment with cathepsin S inhibitors reduced the size of atherosclerotic plaques by 36% in male and 68% in female mice (102). Moreover, cathepsin S selective inhibitors LY3000328 or MIV-247 attenuated myocardial I/R injury by inhibiting inflammation and apoptosis and improved survival at 21 days after ischemic insult, suggesting the potential value of clinical application of cathepsin S inhibitors for cardioprotection (103). However, some studies also reported that cathepsin S deficiency exacerbated LV dilatation and dysfunction after MI, and administration of the non-selective histone inhibitor E64d at 7 and 28 days after MI enhanced inflammatory response and worsened LV dysfunction. Meanwhile, it also inhibited phosphorylation of Smad 2/3, the key molecules of fibrosis signaling pathway, resulting in impaired cardiac remodeling (104). Therefore, whether cathepsin S is involved in pathophysiological alterations in cardiovascular disease by affecting CD74 remains to be further investigated.

### 4.2. Small interfering RNAs (siRNAs)

Small interfering RNAs (siRNAs) are short double-stranded RNAs that can be dissociated into single strands and bound specifically to target messenger RNA (mRNA) sequences. Their conjugation induces target mRNA cleavage and degradation, thus preventing translation and protein expression (105, 106). In recent years, siRNA-based therapeutics have been a popular topic, with numerous medications approved to access the pharmaceutical market (105, 107). CD74 siRNA, which silences the CD74 gene in dendritic cells, may improve dendritic cell-based antitumor immunity (108, 109). CD74 siRNA has been proved to inhibit a variety of tumors, such as breast cancer (110), acute myeloid leukemia (111), head and neck squamous cell carcinoma (112), and brain tumorigenesis (113). Interestingly, CD74 siRNA inhibits atherosclerosis by reducing interferon-gamma (IFNγ)-induced NF-κB activation and monocyte chemoattractant protein-1 (MCP-1) production in human vascular smooth muscle cells, and preventing vascular smooth muscle cells activation (49). Stem/progenitor cell-based treatments are effective therapies for a variety of heart diseases, including ischemic heart disease and heart failure. These cells stimulate angiogenesis and endogenous stem cell activation, thereby improving cardiac function (114, 115). MIF promotes cardiac stem cell survival and proliferation through CD74 to activate the PI3K/Akt/mTOR and AMPK signaling pathways. However, siRNA for CD74 reduced the proliferation of MIF-treated cardiac stem cell, and cell cycle analysis also showed that CD74 knockdown significantly reduced the number of S-phase cells. The activation of AMPK by MIF was significantly inhibited after knockdown of CD74 (115). As a result, since CD74 plays different roles in various cardiovascular diseases, CD74 siRNA may have clinical applications in certain cardiovascular diseases.

### 4.3. Monoclonal antibody of CD74, Milatuzumab

Leukocyte differentiation antigen 74 is highly expressed in a variety of tumor cells, such as breast cancer (116), lymphoma (117), multiple myeloma (118), and chronic lymphocytic leukemia (119, 120). The profile of CD74 expression in tumor cells and its rapid endocytosis properties make it a promising therapeutic target. Monoclonal antibodies are highly uniform antibodies produced by B-cells clones, which only target a specific antigen epitope. They can specifically recognize cell surface markers to achieve precise targeted therapy. Milatuzuma is a synthetic monoclonal antibody that targets CD74 and has been used in the therapy of several tumors, such as chronic granulocytic leukemia (119) and various lymphomas (120). Milatuzuma reduces expression of adhesion molecules β7-integrin and CD62L and inhibits B-cells proliferation (121). In addition, Milatuzumab is also used in the treatment of autoimmune diseases such as SLE (122). Current clinical information on utilization of Milatuzumab in a variety of oncological as well as autoimmune diseases will facilitate the clinical transition of targeted CD74 therapy to cardiovascular disease.

## 5. Conclusion

Leukocyte differentiation antigen 74 is involved in antigen presentation as a molecular chaperone of MHC II and has also been recently identified to be involved in antigen presentation of MHC I. CD74 binds to the two main ligands MIF/MIF2 and activates several downstream signaling pathways. Activation of NF-κB pathway by MIF/MIF2-CD74 promoted B-cells maturation and activation of AMPK pathway promoted glucose uptake and utilization in the myocardium under ischemic and hypoxic conditions as well as pressure overload, which played a protective role for the heart. CD74 also regulates cytosolic calcium homeostasis that plays a regulatory role in cardiac arrhythmias. MIF-CD74 works with CXCR2 to act as a chemotactic agent, promoting atherosclerosis progression. sCD74, a truncated form of the extracellular domain of CD74, was first identified in autoimmune liver disease and subsequently in patients with heart failure and MI, yet its specific role in disease progression remains to be further explored.

Although MIF/MIF2 both play important roles in various diseases through CD74, interaction between MIF/MIF2 and CD74 may be independent. Therapeutic strategies targeting CD74 may provide additional options for the clinical treatment of multiple cardiovascular diseases. The characteristics of high expression of CD74 in atherosclerosis and acute MI and the rapid endocytosis of CD74 provide an essential basis for the precise targeting of CD74 for treatment. Currently, Milatuzumab, an anti-CD74 monoclonal antibody, has been tested in numerous phase I-II clinical trials in a variety of tumors as well as autoimmune diseases. Cathepsin S inhibitors and siRNA targeting the regulation of CD74 function have also made progress in animal models of atherosclerosis or cardiac I/R injury. Nevertheless, the role of CD74 in cardiovascular disease remains obscure and needs to be further studied. Targeted drug therapies for CD74 in cardiovascular disease remain in urgent need of exploration.

## Author contributions

Q-LL drafted the manuscript. JT and LZ generated the images. AR and X-FS edited the manuscript. X-MG revised the manuscript. All authors have read and approved the content of the manuscript.
